# The systemic effect of prolonged use of sodium alendronate on the peri-implant bone repair process

**DOI:** 10.1590/1678-7757-2025-0085

**Published:** 2025-09-29

**Authors:** Paula Buzo FRIGÉRIO, Daniel Silva LEÃO, Pedro Henrique Silva GOMES-FERREIRA, Naara Gabriela MONTEIRO, Daniele BOTTICELLI, Roberta OKAMOTO

**Affiliations:** 1 Universidade Estadual Paulista Faculdade de Odontologia Departamento de Diagnóstico e Cirurgia Araçatuba SP Brasil Universidade Estadual Paulista (UNESP), Faculdade de Odontologia, Departamento de Diagnóstico e Cirurgia, Araçatuba, SP, Brasil.; 2 Universidade Estadual Paulista Faculdade de Odontologia Departamento de Ciências Básicas Araçatuba SP Brasil Universidade Estadual Paulista (UNESP), Faculdade de Odontologia, Departamento de Ciências Básicas, Araçatuba, SP, Brasil.; 3 Centro Universitário de Ourinhos Departamento de Medicina Integrada Ourinhos SP Brasil Centro Universitário de Ourinhos (UNIFIO), Departamento de Medicina Integrada, Ourinhos, SP, Brasil.; 4 ARDEC Academy Rimini Italy ARDEC Academy, Rimini, Italy.

**Keywords:** Dental Implants, Bisphosphonates, Alendronate Sodium, Bone Tissue

## Abstract

Bisphosphonates are the most widely marketed drugs in the world to treat osteoporosis but their anti-remodeling effect can cause osseointegration to fail.

**Objective:**

this study aimed to evaluate bone quality during peri-implant repair in osteopenic rats, adopting an approach inverse to the conventional one in which prolonged treatment with alendronate sodium was initiated only after the installation of titanium implants.

**Methodology:**

implants were installed in the tibias of 32 rats 14 days after ovariectomy (osteopenia). After 14 days, systemic treatment was initiated by gavage with a saline solution or alendronate. At 28 and 56 days after implantation, the rats received calcein (20 mg/kg, i.m.). They were administered alizarin red (20 mg/kg, i.m.) seven days before euthanasia. Euthanasia occurred in two periods, 42 and 70 post-implantation days.

**Results:**

the highest implant removal torques were observed for the OVX ALE at 70 days (13.35 Ncm) and the OVX SAL at 42 days (11.63 Ncm). The expression of bone remodeling and resorption proteins (OPG/ RANKL) was higher in the alendronate-treated animals. In contrast, osteocalcin and bone sialoprotein were similar between the control and alendronate-treated groups. No parameter showed statistically significant variations (BV, BV.TV, Tb.Th, Tb. N, Tb.Sp, and i.S), with similar measurements between groups. Fluorochrome analysis showed active bone remodeling at the implant interface.

**Conclusion:**

the alendronate treatment stabilized the bone-implant interface over time, suggesting a protective effect against osteoporotic bone loss despite a delayed initial response. In the long term, the group systemically treated with alendronate showed improved bone formation around the implants, outperforming the control group. However, future research is essential to prevent possible negative impacts on osseointegration in patients taking this medication.

## Introduction

Osseointegration has promoted a major advance in oral rehabilitation treatment, with a prognosis of success close to 100%, giving patients back their chewing function, aesthetics, and self-esteem.^1^

However, the installation of implants can be impaired due to an imbalance in bone homeostasis, which leads to conditions that are unideal for their fixation.^2^ Patients’ systemic condition configures a factor that directly interferes with the quality of the bone tissue that will form the basis for the installation of implants.^3^ Osteoporosis is a pathology that impairs the trabecular structure, damaging bone mass and resistance and interfering with bone formation around the implant.^2,4^

In women aged over 50 years, estrogen deficiency in the postmenopausal phase imbalances bone remodeling, resulting in resorption that exceeds bone formation due to an increase in osteoclast activity and a decrease in their apoptosis.^5^ Since a large proportion of the female population seeks oral rehabilitation via implants, quality bone formation must occur in direct contact with the endosteal surface of the implant (osteogenesis) to promote primary stability.^6^

In this way, pharmacological therapies have proved to be very effective in controlling postmenopausal osteoporosis. The indicated drugs are classified as antiresorptive (which inhibit osteoclastic activity) and bone-forming agents (which favor bone neoformation). Bisphosphonates, denosumab, and selective estrogen receptor modulators comprise the group of antiresorptive agents,^7-9^ whereas teriparatide and vitamin K belong to the group of bone-forming agents.^10^

Despite a wide range of medications, bisphosphonates are considered the first choice for anti-osteoporosis therapy and the prevention of bone fractures.^10-12^ Among the most widely used drugs, alendronate sodium remains the first choice worldwide^11,12^ either because it is easy to acquire since it is provided free of charge in many countries^13^ or by simple adherence due to its simple administration (orally, weekly), making it an essential choice.^13^

The anti-resorptive efficacy and anti-remodeling effect of bisphosphonates are well-established in the literature, as are possible adverse effects that can interfere with dentistry, such as implantology, which can cause osteonecrosis of the jaws.^14,15^ Its exclusively antiresorptive effect rapidly inhibits bone neoformation increasing bone resorption and impairing bone renewal and turnover around implants.^16^

Considering that our research group has experience with experimental models *in vivo* (rats), evaluating bone formation in peri-implant defects under drug treatment instituted before implant placement,^17,18^ a new clinical question currently arises: the effects of drug therapy initiated after implant placement. This concern is reinforced by clinical studies such as those by Pogrel and Ruggiero (2018)^19^ and Goss, et al.^20^ (2010), who reported significant loss of previously osseointegrated implants in patients who started using bisphosphonates after implant-supported rehabilitation.

Unlike most studies in the literature, which install implants after the start of bisphosphonate therapy, this study proposes an inverse approach, confirmed in the clinical practice of patients who, over the years, may require preventive treatment for osteoporosis with the use of bisphosphonates.

Therefore, this study aimed to assess whether the titanium implant installed after the induction of osteopenia and before the start of systemic treatment with bisphosphonates remained osseointegrated, maintaining the characteristics of good-quality bone tissue, and whether the use of oral bisphosphonates avoided impairing peri-implant repair in the long term.

## Methodology

### Animals and ethical statement

After being accepted by the Ethics in Animal Experimentation Committee (CEUA: 0478-2021), this research began, following the rules established by the ARRIVE Guidelines.^21^ Adult female rats (*Rattus norvegicus Albinus*, Wistar) that were aged three months and weighed about 300 grams, were used from the Araçatuba School of Dentistry—UNESP central animal house.

### Sample size calculation

A power analysis was used to determine the sample size for each group in this study on the tool available at http://www.openepi.com/SampleSize/SSMean.htm (OpenEpi, version 3) based on data obtained from previous studies.^17^ For this calculation, 42.44 and 33.37 means and 8.53 and 4.33 standard deviations were considered, respectively, adopting a 5% significance level and a 95% power for a unilateral hypothesis test. The sample size generated by the digital calculator was n=4 animals per group. A total sample of 32 animals was used in this study, which were distributed into two experimental groups with two euthanasia moments (comprising eight animals per group) to ensure greater statistical robustness and result reliability (Table 1).

**Table 1:** Design of experimental groups.

Groups	N of rats (Euthanasia 42 days)	N of rats (Euthanasia 70 days)
OVX SAL	8	8
OVX ALE	8	8
	16	16
		Total number of animals: 32
		Total number of implants: 64

Prepared by the author, 2025.

### Randomization and allocation

Animals were identified by a numerical sequence that was entered into Microsoft Office Excel (Microsoft, Redmond, Washington, USA). A person with no involvement in this study performed a simple randomization with a 1:1 allocation ratio for groups. The distribution and allocation of rats for surgeries and drug treatment were performed randomly. The rats (four per cage) were kept in an air-conditioned animal facility with a 12-hour light/dark cycle, and received water and food *ad libitum*.

### Experimental groups and study design

**Figure 1:**
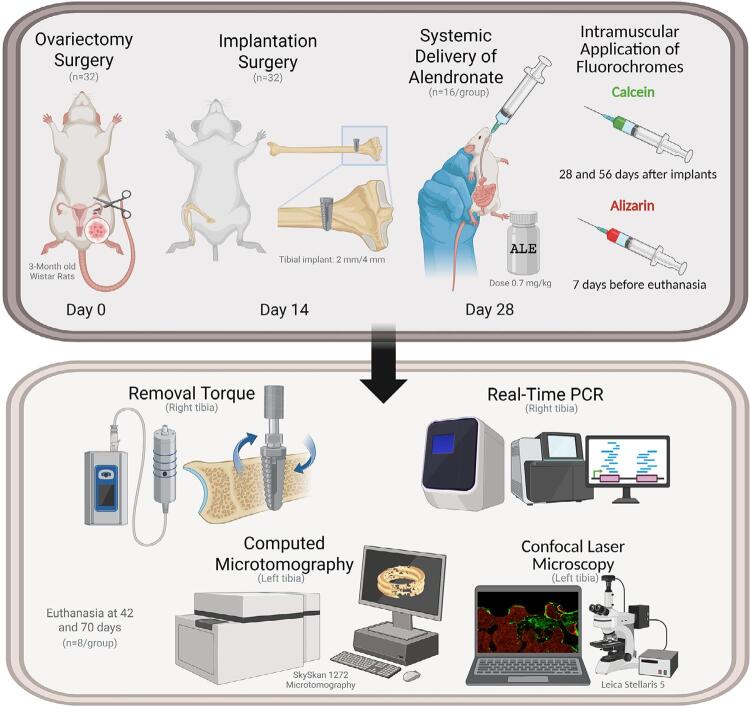
Timeline: Surgical and laboratory procedures. Created by the author with https://www.biorender.com/

In total, 32 rats were divided according to osteoporosis induction surgery and systemic medication: OVX SAL: rats subjected to bilateral ovariectomy and treated with a saline solution (n=16) and OVX ALE: rats subjected to bilateral ovariectomy and treated with sodium alendronate (n=16). Conventional implants were installed in the right and left proximal tibial metaphyses 14 days after castration. Systemic gavage was initiated 14 days later. Then, 16 rats were sacrificed at 42 days (n=8 per group) after implant installation, and the remaining 16 at 70 days after the procedure (n=8 per group). The experiments were conducted in a laboratory to study mineralized tissues, performing biomechanical analyses (removal torque), real-time polymerase chain reaction (RT-PCR), micro-computed tomography (Micro-CT), and laser confocal microscopy (Figure 1).

### Estrous cycle

The rats that were included in the experiment were housed individually subjected to a daily vaginal lavage with one to two drops of saline solution to confirm that they were in a normal estrous cycle. The collected material was aspirated and transferred to histological slides for microscopic evaluation to find the four distinct phases of the estrous cycle.^22^ Only rats with 2-3 regular estrous cycles were included in this study. This technique was performed once more to test the effectiveness of ovariectomy.

### Castration surgery (ovariectomy)

Initially, the rats were anesthetized according to the manufacturer’s recommendations: 5 mg/kg xylazine hydrochloride (Dopaser - Laboratório Calier do Brazil Ltd., Osasco, São Paulo, Brazil) and 50 mg/kg ketamine intramuscularly (Vetaset - Fort Dodge Health Ltd., Campinas, São Paulo, Brazil).^23^ The rats were immobilized on a surgical table in lateral decubitus, the part of the abdomen to be accessed was trichotomized with a 1-cm incision on the flanks, divulsion by planes of the subcutaneous tissue, and of the peritoneum to access the abdominal cavity. The uterine horns were tied with Polyglactin 910 4.0 thread (Vicryl™, Johnson & Johnson, New Brunswick, NJ, USA), and the ovaries were removed bilaterally. The suture was performed in layers with Polyglactin 910 4.0 thread (Vicryl™, Johnson & Johnson, New Brunswick, NJ, USA).^24^

### Implant installation surgery

After 14 days of ovariectomy, the animals were anesthetized using a mixture of ketamine (50 mg/kg) and xylazine hydrochloride (5 mg/kg), following the same anesthetic protocol used for castration procedures.^23^ The medial surface of the right and left tibias was shaved for surgical access. The region was then disinfected using 10% polyvinylpyrrolidone-iodine solution (PVPI; Riodeine, Rioquímica, São José do Rio Preto, SP, Brazil). Using a No. 15 scalpel blade (Feather Industries Ltd., Tokyo, Japan), an incision approximately 1.5-cm long was made over the metaphyseal region of the tibia. Soft tissues were carefully separated and elevated with periosteal elevators to expose the bone surface for implant placement.^17^

A total of 64 commercially available grade IV titanium implants were inserted into the 32 animals, with one implant placed in each proximal metaphysis of both tibias. The implants (TitaniumFix, Tecnologia Componentes Especiais Ltda., São José dos Campos, SP, Brazil) featured a surface modified by dual acid etching using nitric, hydrofluoric, and sulfuric acids, measured 2 mm in diameter and 4 mm in height, and were sterilized via gamma irradiation. Both bone cortices were drilled using 1.6- and 2.0-mm helical burs, fitted to a contra-angle handpiece (3624N 1:4, head 67RIC 1:4, KaVo^®^, Kaltenbach & Voigt GmbH & Co, Biberach, Germany), and operated by an electric motor (BLM 600^®^; Driller, São Paulo, SP, Brazil) at 1000 rpm, with continuous irrigation using 0.9% isotonic saline solution (Fisiológico^®^, Laboratórios Biosintética Ltd., Ribeirão Preto, SP, Brazil). Each rat received two implants per limb, totaling two implants in each metaphyseal site. The surgical site was closed using absorbable sutures (Polyglactin 910 – Vicryl 4.0, Ethicon, Johnson & Johnson, São José dos Campos, SP, Brazil) for the internal layer and non-absorbable monofilament sutures (Nylon 5.0, Ethicon, São José dos Campos, SP, Brazil) externally.^17^

Immediately after surgery, animals were administered an intramuscular injection of 0.2 mL of penicillin G benzathine (Pentabiotic Veterinário Pequeno Porte, Fort Dodge Saúde Animal Ltd., Campinas, SP, Brazil) for infection prophylaxis.^25^

### Systemic drug treatment with alendronate sodium

Alendronate sodium (Boraschi Vieira Ribas & Cia Ltd., Araçatuba, São Paulo, Brazil) was weekly administered at a dose of 0.7 mg/kg according to the instructions on its package leaflet by gavage using a curved needle (Insight equipamentos, Ltd., Ribeirão Preto, SP, Brazil).^18,26^ It was administered 14 days after the surgery to install the implants and was maintained throughout the experiment until the animals were euthanized. The final volume of alendronate sodium administered totaled 0.3 mL. The animals in the SAL (saline solution) group received 0.3 mL of 0.9% saline solution every week to undergo the same stress as those in the ALE (alendronate sodium) group.

### Application of fluorochromes

After 28 and 56 days of implant installation surgery, rats from the first and second euthanasia periods received an intramuscular injection of fluorochrome calcein (20 mg/kg) and one week (7 days) before euthanasia the same animals from both euthanasia periods received an intramuscular injection of fluorochrome alizarin red (30 mg/kg), respectively.^27^

### Euthanasia

Euthanasia was performed at two time points: 16 animals were euthanized 42 days after implant placement, whereas the remaining ones, 70 days after implantation. Thus, each group (OVX SAL and OVX ALE) consisted of eight animals per euthanasia interval. The animals were first anesthetized, and biomechanical testing (removal torque) was performed on their right tibia, along with bone tissue collection for RT-PCR analysis. Subsequently, these animals received a lethal dose of anesthetic to enable the extraction of the left tibia, which was used for Micro-CT and confocal laser microscopy analysis.

### Performed analysis

#### Biomechanical Analysis (Removal Torque)

A specific implant mount (Neodent, Ltd., Curitiba, Paraná, Brazil) was connected to a digital torque device and fitted into the hexagonal interface of the implant. A counterclockwise rotation was gradually applied to increase the reverse torque, rotating the implant within the surrounding bone matrix and disrupting the bone–implant connection. The peak torque required to cause this failure was recorded in Newton centimeters (Ncm) using the torque device.

## RT-PCR analysis

Following the reverse torque procedure, the right tibiae of the animals were surgically accessed, and the bone in direct contact with the implant threads was carefully extracted using the appropriate forceps. The bone specimens were rinsed in phosphate-buffered saline, rapidly frozen in liquid nitrogen, and stored at –80 °C. Total RNA was isolated using Trizol reagent (Life Technologies: Invitrogen, Carlsbad, CA, USA). RNA samples were assessed for quality, purity, and concentration and subsequently reverse-transcribed into cDNA using M-MLV reverse transcriptase (Promega Corporation, Madison, WI, USA). The generated cDNAs were combined with Taqman Fast Advanced Mastermix (Applied Biosystems) and added to a 96-well PCR plate (Life Biotechnologies) optimized for fast thermal cycling. Gene expression was evaluated using Taqman Gene Expression Assays, targeting genes related to bone repair. The analyzed genes included osteoprotegerin (OPG), receptor activator of nuclear factor kappa-B ligand (RANKL), osteocalcin, and bone sialoprotein (Table 2). A quantitative real-time PCR was conducted to measure the expression levels of markers associated with bone remodeling, resorption, and formation.

**Table 2:** TaqMan probes for real-time PCR.

Gene	Coding Gene	Reference Gene
OPG	Tnfrsf1	Rn00563499_m1
RANKL	Tnfsf11	Rn80589289_m1
OCN	BGGLAP	Rn00566386_g1
IBSP	IBSP	Rn00561414_m1

## Computerized microtomography (Micro-CT)

The left tibiae were fixed in 10% formaldehyde for 48 hours, washed in water for 24 hours, immersed in 70% alcohol, and scanned using the SkyScan 1272 computerized microtomograph to obtain three-dimensional reconstructed images on NRecon (SkyScan, Leuven, Belgium, 2011; Version 1.6.6.0). The sections were 8-µm thick and used an Al. 0.5- and Cu 0.038-mm filter with a 2016×1344 resolution and a two-hour-and-16-minute acquisition time for each sample. The images were positioned and aligned on Data Viewer (SkyScan, Leuven, Belgium, Version 1.4.4 64-bit). They could be observed in the transverse, longitudinal, and sagittal planes. Next, using CTAnalyser (2003-11 SkyScan, 2012 Bruker MicroCT Version 1.12.4.0), an area around the implant was defined, delimited by 0.5 mm around the entire implant and along the whole length of the reparative bone in the tibial region. This was defined as the total area (4.5 mm × 3.2 mm). CTAnalyser analyzes and measures images according to a grayscale (threshold). In total, 255-80 shades of gray were used as the threshold for the implant and 255-20 ones for the bone to obtain the volume and porosity of the bone formed around the implants. The 3D images were obtained on CTVox (SkyScan, Leuven, Belgium, 2003; Version 3.3.1).

Bone volume (BV), percentage of bone volume (BV.TV), trabecular bone thickness (Tb.Th), trabecular number (Tb.N), trabecular separation (Tb.S), and bone-implant contact (i.S) were assessed according to Bouxein’s Guidelines.^28^

## Laser confocal microscopy

After microtomography analysis, the samples were prepared for analysis by laser confocal microscopy. The tibias were dehydrated in a sequence of increasing alcohol concentrations (70, 80, 90, 95, and 100%). Once dehydration was complete, the pieces were immersed in solutions of absolute alcohol and methyl methacrylate in progressively increasing concentrations. They were then included in methyl methacrylate containing 1% benzoyl peroxide (catalyst) in test tubes and kept in an oven at 60°C for seven days for resin polymerization, after which the samples were sectioned with a Maxicut and sanded with a polisher (PL02E/300 politriz metalográfica de duas velocidades; Equipamentos Metalográficos, São Paulo, SP, Brasil) until sections with a thickness of approximately 80 μm were obtained, which were mounted on slides for analysis. Slides were examined using a Leica Stellaris 5 laser confocal microscope (Stellaris 5; Leica Microsystems, Wetzlar, Germany).

In the analysis, the peri-implant bone showed two fluorochrome markings—calcein (green) and alizarin (red)—corresponding to periods of calcium deposition, evincing the conversion of old bone (calcein) into new bone (alizarin). The images obtained were saved in TIFF format and analyzed on ImageJ (Processing Software and Image Analysis, Ontario, Canada).

**Figure 2:**
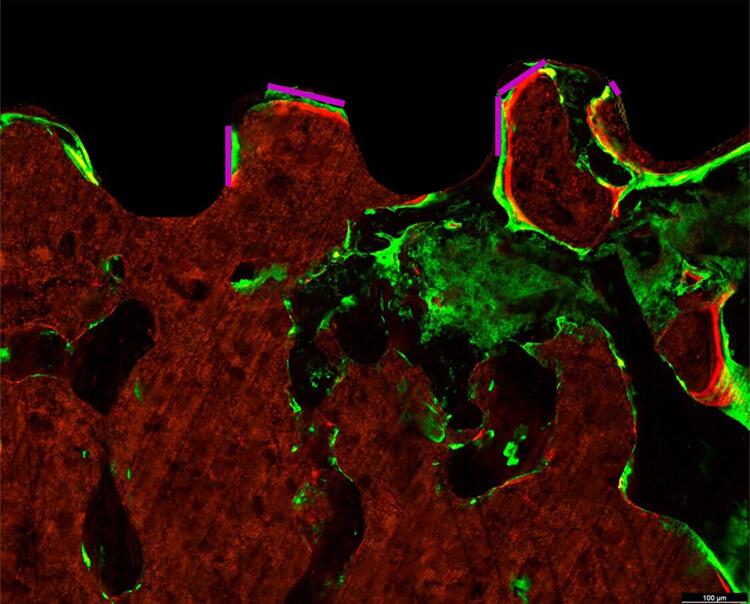
Representation of confocal laser microscopy of the peri-implant region. The figure was standardized for analysis of bone-implant contact (BIC) based on the perimeter delimited by the purple lines. BIC was determined by the green (calcein) and red (alizarin) fluorescent markers deposited at the bone-implant interface, specifically in the region corresponding to the third and fourth turns of the implants.

To quantify the linear length of the bone-implant interface (BIC),^29^ the perimeter was obtained in micrometers from the interfaces of the regions corresponding to the third and fourth turns of the implants. Only the green (calcein) and red (alizarin) fluorescent lines in the peri-implant interface were considered on the “Straight” tool (Figure 2).

## Statistical analysis

The data obtained from the removal torque, RT-PCR, micro-CT, and laser confocal microscopy analyses were subjected to statistical analysis on GraphPad Prisma 8.1.1. The values were tabulated and subjected to the two-way ANOVA test, with Šidák’s multiple comparisons test. Significance level (p<0.05).

## Results

### Removal torque (Ncm)

**Figure 3:**
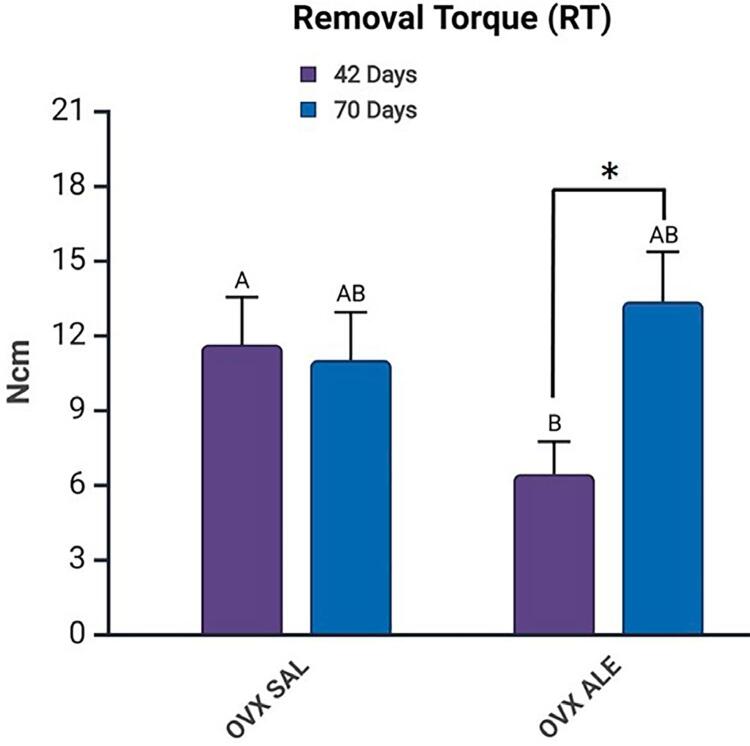
Mean removal torque results. Statistical differences are represented by letters and symbols (A, B,*). Intergroup difference between systemic treatments, SAL vs. ALE (A vs. B), and the intragroup difference between the same systemic treatment and 42 vs. 70 days (*), (p<0.05, two-way ANOVA and Šidák’s multiple comparisons test). Legend: AB without statistical difference.

For the analysis of the removal torque, which considered the statistical difference between the euthanasia period and the systemic treatment (intergroup comparison), a statistically significant difference was observed between OVX SAL 42 days vs. OVX ALE 42 days (Šidák’s multiple comparisons test, p=0.0187, represented by A vs. B). The highest removal torques were seen for the OVX ALE 70 days (13.35 Ncm) and OVX SAL 42 days (11.63 Ncm), with no statistical difference between them. The lowest removal torque was for the OVX ALE 42 days group (6.42 Ncm) (Figure 3).

When comparing the same systemic treatment (intra-group comparison), a statistically significant difference was observed between the groups treated with alendronate sodium, OVX ALE 42 days (6.42 Ncm) vs. OVX ALE 70 days (13.35 Ncm) (Šidák’s multiple comparisons test, p=0,0083, represented by *). At 70 days of euthanasia, the mean removal torque increased for the group treated with alendronate sodium. No statistical difference occurred when compared to the control treatment, OVX SAL 42 days (11.63 Ncm) vs. OVX SAL 70 days (11.0 Ncm) (Figure 3).

### RT-PCR analysis

**Figure 4:**
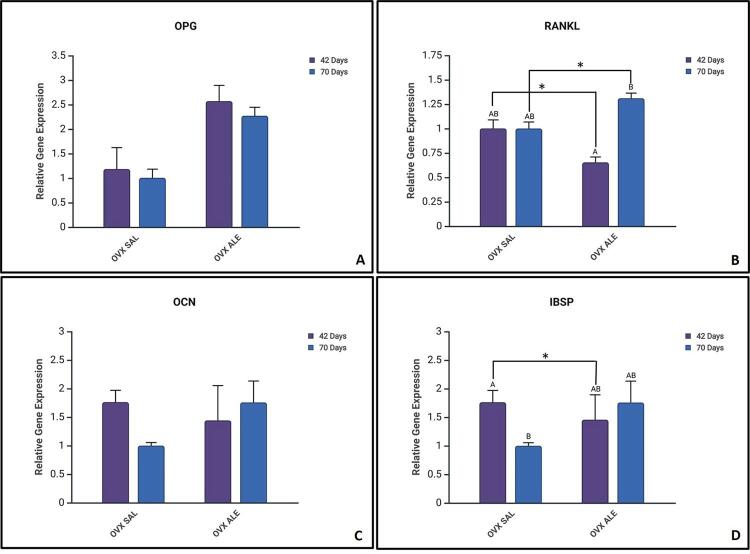
Mean results of RT-PCR analysis, (A) OPG, (B) RANKL, (C) osteocalcin, and (D) bone sialoprotein, with statistical differences represented by letters and symbols (A, B,*). Intragroup difference between the same systemic treatment at 42 vs. 70 days (A vs. B) and intergroup difference between systemic treatments, SAL vs. ALE (*), (p<0.05, two-way ANOVA and Šidák’s multiple comparisons test). Legend: AB without statistical difference.

OPG is a protein that plays a fundamental role in osteoclast biology. The highest expression of this gene was observed in the 42-day OVX ALE group. No statistically significant difference occurred between the groups OVX SAL 42 days (1.174), OVX SAL 70 days (1.007), OVX ALE 70 days (2.569), and OVX ALE 70 days (2.272) (Figure 4A).

RANKL is related to bone regeneration and remodeling, and its receptor is OPG. The highest expression of this gene was observed in the OVX ALE 70 days group (1.293). The OVX ALE 42 days vs. OVX ALE 70 days groups showed a statistically significant intragroup difference (0.6532) (Šidák’s multiple comparisons test, p=0.0036, represented by A vs. B) but control, OVX SAL 42 days (1.001), and OVX SAL 70 days (0.9991) groups evinced no such difference. The intergroup evaluation showed a statistical difference between OVX SAL vs. OVX ALE 42 days (Šidák’s multiple comparisons test, p=0.0121, represented by*) and OVX SAL vs. OVX ALE 70 days (Šidák’s multiple comparisons test, p=0.0168, represented by *) (Figure 4B).

The gene osteocalcin mineralizes and matures osteoblasts. No statistically significant difference occurred by comparing the groups for this gene, which showed similar results, OVX SAL 42 day (1.759), OVX SAL 70 day (0.9988), OVX ALE 70 day (1.457), and OVX ALE 70 day (1.758) (Figure 4C).

Bone sialoprotein is an essential component of bone tissue mineralization. An intragroup statistical difference was observed between the OVX SAL 42 days (1.412) vs. OVX SAL 70 days (0.9972) groups (Šidák’s multiple comparison test, p=0.0041, represented by A vs. B), with higher values for the bone sialoprotein gene at 42 days. OVX ALE 42 days (0.9244) and OVX ALE 70 days (1.038) showed no statistical difference. The intergroup evaluation evinced a statistical difference between OVX SAL vs. OVX ALE 42 days (Šidák’s multiple comparison test, p=0.0041, represented by *) (Figure 4D).

The RANKL-OPG ratio describes the proportional relation between the RANKL and OPG genes, two factors that play an important role in bone metabolism. The OVX SAL 42-day (0.6867) vs. OVX ALE 42-day (1.357) groups showed a statistically significant intergroup difference (Šidák’s multiple comparison test, p=0.0211, represented by A vs. B).

**Figure 5:**
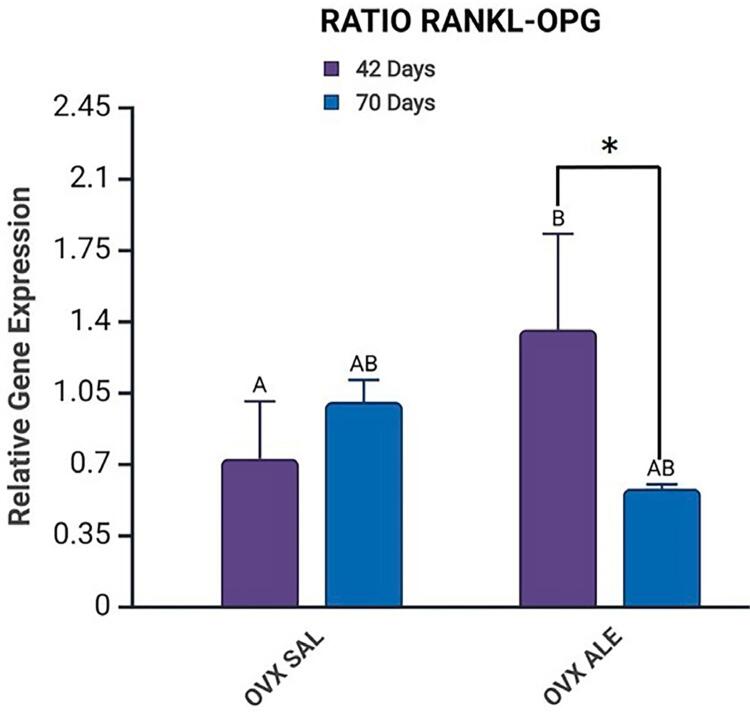
Mean results of RT-PCR analysis for the RANKL-OPG ratio, with statistical differences represented by letters and symbols (A, B,*). Intergroup difference between systemic treatments, SAL vs. ALE (A vs. B), and intragroup difference between the same systemic treatment at 42 vs. 70 days (*), (p<0.05, two-way ANOVA and Šidák’s multiple comparisons test). Legend: AB without statistical difference.

In the intragroup comparison, a statistically significant difference was observed between the alendronate sodium-treated groups, OVX ALE 42-day vs. OVX ALE 70-day (Šidák’s multiple comparison test, p=0.0155, represented by *). The control groups showed no statistically significant difference (Figure 5).

### Computerized microtomography (Micro-CT)

When comparing the first euthanasia period with the second, 6S vs. 10S, respectively, 42 and 70 days, no statistical difference was observed for any parameter (ANOVA, p>0,05).

**Figure 6:**
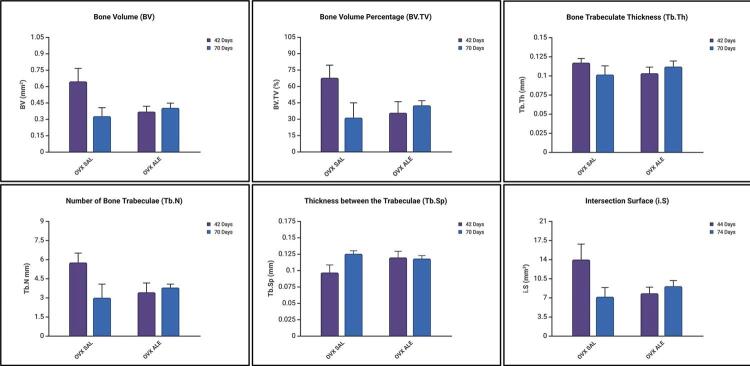
Mean micro-CT results with no statistical differences between the 42- vs. 70-day periods (p>0.05, two-way ANOVA and Šidák’s multiple comparisons test).

The 42-day OVX SAL group showed greater bone volume (BV=0.64547 mm^3^), percentage of bone volume (BV.TV=67.704 %), thickness between bone trabeculae (Tb.Th=0.11708 mm), number of trabeculae (Tb.N=5.759 mm), and bone-implant contact as assessed by the intersection surface (i.S=13.974 mm^3^). The group showed the lowest separation parameter between the trabeculae (Tb.Sp=0.09671 mm) (Figure 6).

The OVX SAL 70 days and OVX ALE 42 days groups had the worst average for bone volume and percentage, thickness, number of trabeculae, and intersection surface, showing greater space between the bone trabeculae. At 70 days, the animals treated with alendronate showed improved results (BV=0.403 mm^3^; BV.TV=42.44 %; Tb.Th=0.1119 mm; Tb.N=3.792 mm; Tb.Sp=0.1179 mm and i.S=9.094 mm^3^) with no statistical difference with the first period. (Figure 6).

**Figure 7:**
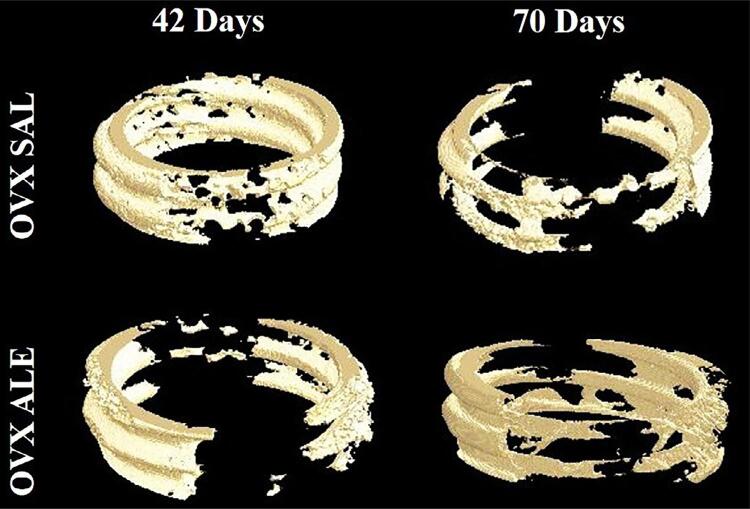
Three-dimensional reconstruction (CT Vox) of the formed bone repair representing the evaluated groups.

The three-dimensional CT Vox software reconstruction showed less bone formation around the titanium implant spirals in the OVX SAL 70 days and OVX ALE 42 days groups. At 42 days, the OVX SAL group showed better peri-implant bone repair, as did the group treated systemically with long-term sodium alendronate (Figure 7).

### Laser confocal microscopy (Bone-implant interface)

Confocal laser microscopy analysis found two distinct layers of fluorochromes, calcein (old bone) and alizarin (new bone) at BIC, indicating sequential calcium deposition and new bone formation.

At 42 days, the OVX SAL group showed the highest mean BIC (364,825 µm), with a statistically significant difference to the OVX ALE group at 42 days (285,080 µm) in the intergroup comparison (Šidák’s multiple comparison test, p=0.0059, represented by A vs. B) During this period, osseointegration was more efficient in the absence of the alendronate sodium treatment. At 70 days, the OVX SAL (278,788 µm) and OVX ALE groups (273,597 µm) showed intermediate values with no statistical differences.

**Figure 8:**
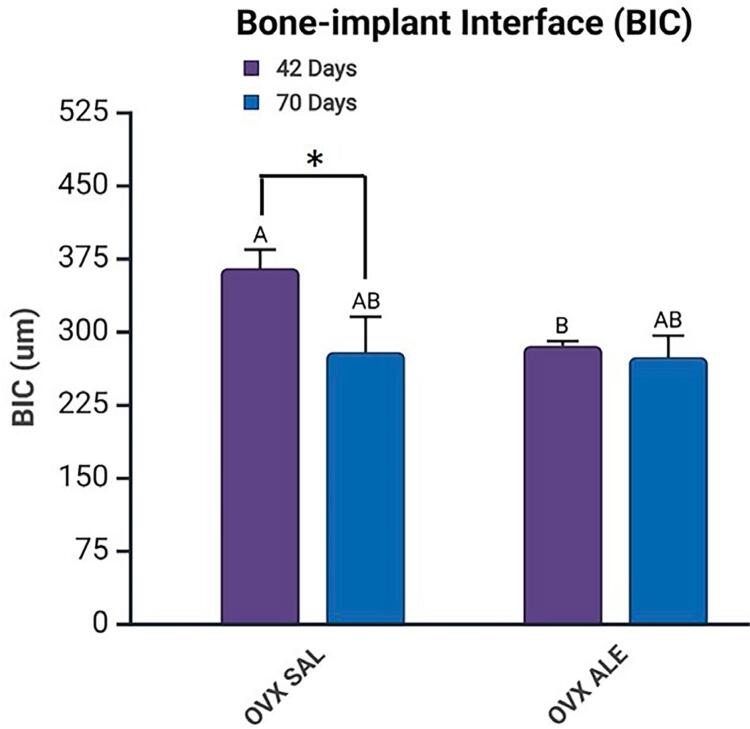
Mean bone-implant interface (BIC) results with statistical differences represented by letters and symbols (A, B,*). Intergroup difference between systemic treatments, SAL vs. ALE (A vs. B), and intragroup difference between the same systemic treatment at 42 vs. 70 days (*), (p<0.05, two-way ANOVA and Šidák’s multiple comparisons test). Legend: AB without statistical difference.

Comparing the same systemic treatment and the different periods, 42 vs. 70 days (intragroup comparison) found a statistically significant difference between OVX SAL 42 days vs. OVX SAL 70 days (Šidák’s multiple comparison test, p=0.0038, represented by *). This suggests that the absence of alendronate decreased osseointegration from 42 to 70 days, possibly associated with the advancement of bone resorption resulting from the osteoporosis caused by ovariectomy (Figure 8).

## Discussion

According to the International Osteoporosis Foundation, women are the most affected by osteoporosis, as about 21.2% of them are impacted by it.^30,31^ Osteoporosis also delays the osseointegration of titanium implants in humans and animals.^31-33^ Currently, many patients seeking surgical treatment for implants are older women affected by osteoporosis, primarily facing issues such as tooth loss or total edentulism.^34^ Research in progress seeks therapies that stimulate quality bone formation and preserve osseointegration.

The experimental design in Ramalho-Ferreira, et al.^18^ (2015) evaluated the systemic medication applied before implantation. In their protocol, alendronate showed no significant effect on peri-implant bone repair. In this study, alendronate therapy was initiated after implant placement to assess whether systemic treatment would compromise osseointegration, differing from the findings of Ramalho-Ferreira, et al.^18^ (2015) since clinical evidence suggests that initiating bisphosphonate use after implant placement may compromise the maintenance of osseointegration.

Marx, et al. (2005)^35^ reported cases of osteonecrosis of the jaw and implant failures in patients treated with intravenous bisphosphonates. Migliorati, et al. (2005)^36^ discussed the theoretical risk of these complications in oral bisphosphonate users, although without direct clinical evidence of failure. Furthermore, the World Health Organization guidelines acknowledge this risk (although lower with oral bisphosphonates).^37^ These findings highlight the importance of experimental models that evaluate the effects of late administration of these drugs, such as in this study.

Animals treated with sodium alendronate showed low removal torque in a short period—OVX ALE 42 days (6.42 Ncm). However, in the long term this condition improved—OVX ALE 70 days (13.35 Ncm)—, making it possible to understand that its inhibitory effect on osteoclastic activity was essential to release calcium into the bloodstream, reducing bone resorption.^38^ The control group (OVX SAL) maintained similar torque values between the evaluated periods without statistical differences, which suggests an early stabilization of osseointegration comparable to that in the group treated with alendronate for a long time.

Regarding gene expression analysis, RANKL plays a critical role in regulating the formation and activity of osteoclasts, the cells responsible for bone resorption. Its main function is to differentiate and activate osteoclasts.^39^ The highest expression of this gene was observed in the OVX ALE 70-day group (1.293), with a statistical difference to the OVX ALE 42-day group (0.6532). However, the balance between RANKL and OPG is crucial for bone homeostasis since it is an antagonist of bone resorption.^40^ Thus, both groups had the highest expressions for the OPG gene—OVX ALE 42-day (2.569) and OVX ALE 70-day (2.272)—with no statistically significant difference. The control group (OVX SAL) showed no statistical difference from 42 (1.001) to 70 days (0.9991).

In the analysis of the ratio between RANKL and OPG, the OVX ALE 42-day group showed higher values (1.357), with a statistical difference in relation to the OVX SAL 42-day group (0.6867), indicating an initial imbalance favorable to bone resorption. Alendronate sodium may initially increase bone resorption by interfering with osteoclastic activity but shows an antiresorptive and stabilizing effect on bone remodeling over time, as per the reduction of the RANKL/OPG ratio. In contrast, in the untreated group (OVX SAL), the opposite pattern was observed: increased bone resorption in the long term, consistent with the progression of osteopenia induced by ovariectomy.

Osteocalcin is produced by osteoblasts and plays a role in mineralization, favoring calcium deposition.^41^ Results were similar across groups, with no statistical differences. Bone sialoprotein is essential for bone formation and mineralization, playing a fundamental role in cell adhesion and the organization of the extracellular matrix.^42^ The OVX SAL group showed a significant reduction from 42 to 70 days, indicating a decrease in osteoblastic activity over time. In contrast, the animals treated with alendronate maintained stable levels, suggesting a modulating effect of the drug. The intergroup comparison at 42 days showed lower expression in the treated group, reinforcing the initial impact of alendronate on osteoblastic activity.

As with the removal torque results, microtomography showed that the 42-day OVX ALE group had the lowest values for volume, bone percentage, trabecular thickness, and number and greater trabecular spacing, indicating lower bone quality. At 70 days, the slight improvement with no statistically significant difference. Alendronate neither compromised the osseointegration of implants nor promoted greater bone formation than the control, which is consistent with the experimental model of osteopenia.

At 42 days, the OVX SAL group showed the best results for bone volume, bone percentage, thickness, and number of trabeculae and the largest bone-to-implant contact surface. However, the untreated animals showed imbalanced bone remodeling over time, with inferior results for all evaluated parameters. It is worth noting that the osteopenic condition of the rats impacted these results.

Fluorochrome analysis confirmed new bone formation at the bone-implant interface, showing dynamic remodeling even under compromised systemic conditions. In an osteoporotic model, osseointegration was influenced by time and alendronate treatment. The more favorable initial bone response in the untreated group suggests that physiological bone remodeling was more active in the early stages, favoring bone-implant contact. However, the significant reduction in BIC over time in this same group indicates that osseointegration tends to deteriorate in the absence of pharmacological intervention, possibly due to the advancement of bone resorption associated with estrogen deficiency.

Conversely, although the alendronate-treated group initially had a lower BIC, values remained stable over time, suggesting a delayed protective effect of the drug on peri-implant bone. This stabilization may be related to the inhibition of osteoclastic activity by the bisphosphonate, which, although it can delay initial bone formation, contributes to preserving bone matrix in the medium term.

The results of this study are consistent with previous research on the positive effect of alendronate on bone repair around implants in rats with induced osteoporosis, with improvement evinced by microtomographic and molecular analyses.^26^

Alendronate sodium is a bisphosphonate commonly prescribed to treat osteoporosis and other conditions that affect bone density.^43,44^ Evidence indicates that prolonged use of alendronate promotes bone formation and increases bone mineral density in the long term.^44^ However, its prolonged use can compromise bone quality and turnover,^45^ highlighting that and bone quantity, quality, and turnover are essential for the stability and longevity of implants.

Unlike Ramalho-Ferreira, et al.^18^ (2015), which reported impaired osseointegration when alendronate was present at the time of implant placement, the data from this study suggest that delayed initiation of therapy may repair bone without compromising implant integration. The findings of this research corroborate previous studies on the beneficial effects of bisphosphonates on peri-implant bone repair in osteoporotic models. However, questions remain regarding the quality of the newly formed bone tissue.^46,47^ In particular, it is noteworthy that the experimental protocol in this study, with alendronate use beginning after implant placement, simulates a relevant clinical condition given that many patients initiate anti-osteoporosis treatment after implant-supported rehabilitation.

Histological analyses are important to detail information on inflammation, vascularization, and bone matrix formation. We acknowledge that the absence of these analyses constitutes a limitation of this study. However, this initial investigation aimed to evaluate the long-term effects of systemic medication after implant placement, prioritizing bone mineralization parameters. It is also worth noting that, unlike experimental models with zoledronic acid, which induces more pronounced inflammatory and vascularization responses, we chose to use alendronate sodium, a drug with lower pharmacological potency and greater widespread availability. This choice was chosen to investigate more subtle effects that clinically represent the reality of most osteoporotic patients exposed to this medication. In future studies within this context, we plan to include these histological analyses to complement and deepen the obtained findings.

Results show that the osteopenia induced by ovariectomy was not spontaneously reversed as the control group had poor long-term peri-implant bone formation. In contrast, alendronate administration improved this bone response without compromising implant osseointegration. However, it is important to emphasize that the prolonged use of bisphosphonates still raises questions, and further investigations are needed to explain their effects on bone dynamics and the stability of osseointegrated implants.

## Conclusion

Over time, the group undergoing systemic treatment with alendronate sodium showed improved bone regeneration around the implants, surpassing the control group (70 days). However, its side effects and prolonged time for elimination from the body must be considered. Therefore, future research is essential to prevent possible negative impacts on osseointegration in patients using this medication.
